# Incubation recess behaviors influence nest survival of Wild Turkeys

**DOI:** 10.1002/ece3.5843

**Published:** 2019-11-21

**Authors:** Nicholas W. Bakner, Landon R. Schofield, Cody Cedotal, Michael J. Chamberlain, Bret A. Collier

**Affiliations:** ^1^ School of Renewable Natural Resources Louisiana State University Agricultural Center Baton Rouge Louisiana; ^2^ Louisiana Department of Wildlife and Fisheries Baton Rouge Louisiana; ^3^ Warnell School of Forestry and Natural Resources University of Georgia Athens Georgia; ^4^ Present address: Warnell School of Forestry and Natural Resources University of Georgia Athens Georgia

**Keywords:** incubation behavior, nest success, recess movements, reproduction, Wild Turkey

## Abstract

In ground nesting upland birds, reproductive activities contribute to elevated predation risk, so females presumably use multiple strategies to ensure nest success. Identification of drivers reducing predation risk has primarily focused on evaluating vegetative conditions at nest sites, but behavioral decisions manifested through movements during incubation may be additional drivers of nest survival. However, our understanding of how movements during incubation impact nest survival is limited for most ground nesting birds. Using GPS data collected from female Eastern Wild Turkeys (*n* = 206), we evaluated nest survival as it relates to movement behaviors during incubation, including recess frequency, distance traveled during recesses, and habitat selection during recess movements. We identified 9,361 movements off nests and 6,529 recess events based on approximately 62,065 hr of incubation data, and estimated mean nest attentiveness of 84.0%. The numbers of recesses taken daily were variable across females (range: 1‒7). Nest survival modeling indicated that increased cumulative distance moved during recesses each day was the primary driver of positive daily nest survival. Our results suggest behavioral decisions are influencing trade‐offs between nest survival and adult female survival during incubation to reduce predation risk, specifically through adjustments to distances traveled during recesses.

## Graphical abstract

Identification of drivers reducing predation risk have primarily focused on evaluating vegetative conditions at nest sites, but behavioral decisions manifested through movements during incubation may be additional drivers of nest survival. Using GPS data collected from female Eastern wild turkeys (*n* = 206), and we evaluated nest survival as it relates to movement behaviors during incubation, including recess frequency, distance traveled during recesses, and habitat utilization during recess movements. Our results suggest behavioral decisions are influencing trade‐offs during incubation to reduce predation risk to nests, specifically through distance traveled during recesses.

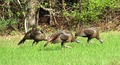

## 1 INTRODUCTION

Annual reproduction is the primary driver of avian population dynamics across a wide variety of species and systems (Martin, 1995). Identifying factors underlying variability in reproductive success is central to improving understanding of population dynamics (Ghalambor & Martin, 2002; Martin, 2002). Reproductive activities are known to be expensive to avian species, resulting in periods of high predation risk, reduced energy acquisition and impacts to embryonic development (Deeming & Reynolds, 2015; Fontaine & Martin, 2006; Skutch, 1962). Hence, birds use a wide array of behavioral strategies during incubation to ensure nest success in dynamic landscapes (Deeming, 2002).

Ground‐nesting upland birds are inextricably linked to nest locations during incubation, which limits foraging opportunities and potentially exposes individuals to elevated predation risk (Deeming & Reynolds, 2015; Skutch, 1962). Evaluations of likely drivers of reproductive success have regularly focused on vegetative conditions at the nest site (Batary & Baldi, 2004; Ghalambor & Martin, 2002; Martin, 1993), as vegetation is thought to mitigate predation risk and influence nest‐site selection (Orians & Wittenberger, 1991). However, the distribution of vegetation and resources around nest sites may have fitness consequences to females (Jones, 2001), which are manifested via behavioral decisions during incubation. Therefore, behavioral activities undertaken during incubation may mitigate risk of nest loss (Deeming, 2002; Martin, 1993).

Incubation recesses are directional movements made away from nesting locations during active incubation, which are thought to allow individual's time to acquire necessary resources (Deeming, 2002) while maintaining appropriate egg temperatures (Deeming, 2002; Fu et al., 2017; Jia, Sun, & Swenson, 2010; Naylor, Szuba, & Bendell, 1988; Webb, 1987). However, movements associated with recesses may increase predation risk to both the female and nest (Martin, 2002), and therefore, the distribution of resources within an accessible landscape during incubation should drive frequency and distance of individual recess movements (Conley et al., 2015). Currently, the consequences of recess movements on nest survival of ground‐nesting upland birds are generally unknown (Jones, 2001; Orians & Wittenberger, 1991), but there is evidence suggesting that activities such as recess bout frequency can impact reproductive effort (Burnam et al., 2012; Coates & Delehanty, 2008; Conway & Martin, 2000; Kessler, 1962; Smith, Tulp, Schekkerman, Gilchrist, & Forbes, 2012; Wiebe & Martin, 1997). Thus, linking behavioral activities during incubation with conditions of the surrounding landscape may provide insight into individual drivers of reproductive success (Aldrich & Raveling, 1983; Dudko, Coates, & Delehanty, 2019; Naylor et al., 1988; Smith et al., 2012).

The Eastern Wild Turkey (*Meleagris gallopavo silvestris*; hereafter, wild turkey) is a ground‐nesting uniparental Galliform widely distributed across the United States and southern Canada. Duration of incubation ranges from 25 to 29 days during March‒July (Healy, 1992) and during incubation, and females are restricted to an incubation range around the nest site (Conley et al., 2015; Healy, 1992). Nest‐site selection and vegetative characteristics at the nest site have historically been considered the primary driver of reproductive success for wild turkeys (Badyaev, Etges, & Martin, 1996; Chamberlain & Leopold, 2000), but contemporary works have noted that vegetative conditions at nest sites may have limited importance to nest success (Byrne & Chamberlain, 2013; Conley et al., 2015; Little, Chamberlain, Conner, & Warren, 2016; Streich, Little, Chamberlain, Conner, & Warren, 2015; Yeldell, Cohen, Little, Collier, & Chamberlain, 2017).

Conversely, behavioral decisions during incubation could underlie population dynamics (Conley et al., 2015), specifically nest survival could be influenced by incubation recess behaviors. Incubation recesses by wild turkeys were believed to be geared toward ensuring that incubating females can defecate and forage away from nest sites (Green, 1982; Martin, Juhan, Palmer, & Carroll, 2015; Williams, Austin, Peoples, & Phillips, 1971) but recesses have rarely been accurately documented in the field (Conley et al., 2015; Williams et al., 1971). Notably, the extant literature on incubation recess behaviors by wild turkeys is based on a small sample of observations of females either leaving or returning to nest sites (Green, 1982; Spohr, 2001; Williams et al., 1971). As incubating females must balance recess movements with increased predation risk, there is potential that recess movements and resources selected by females during recesses could impact nest survival (Conley et al., 2015) and potentially female survival during the breeding period (Collier, Melton, Hardin, Silvy, & Peterson, 2009). Understanding how incubation recess behaviors are related to nest survival is unknown, yet potentially important aspect of our collective understanding of wild turkey reproductive ecology. Our objectives were to (a) describe incubation recess behaviors and evaluate relationships between behavioral activities and nest survival, and (b) evaluate space and habitat use of incubating females at multiple spatial scales around the nest site to determine whether differences in nest success were driven by landcover types within incubation ranges.

## 2 STUDY AREA

**Figure 1:**
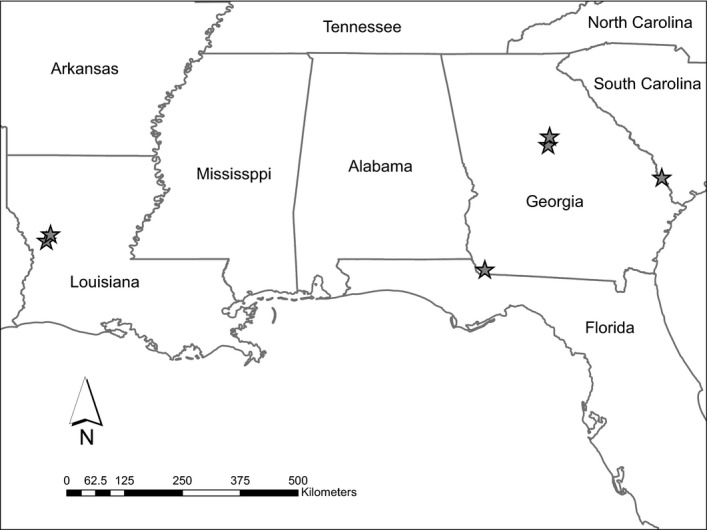
Map of study sites across the southeastern United States where incubation recess behaviors were evaluated for female Eastern Wild Turkeys (*Meleagris gallopavo silvestris*) during 2014‒2017

We conducted research on 6 study sites throughout the southeastern United States (Figure 1). In South Carolina, we conducted research on three contiguous wildlife management areas (WMA; Webb, Hamilton Ridge, and Palachucola; hereafter Webb WMA Complex), all managed by the South Carolina Department of Natural Resources (SCDNR). The Webb WMA Complex was dominated by longleaf pine (*Pinus palustris*), loblolly pine (*P. taeda*), and slash pine (*P. elliottii*) forests with hardwood stands along riparian corridors and expanses of bottomland hardwood wetlands consisting of oaks (*Quercus* spp.). Prescribed fire was applied on an approximately 3‐ to 5‐year return interval. For a detailed description of site conditions on the Webb WMA Complex, see Wightman et al. (2018).

In Georgia, we conducted research on three sites: Silver Lake, B. F. Grant, and Cedar Creek WMA. The Silver Lake WMA, located in southwest Georgia, was owned and managed by the Georgia Department of Natural Resources—Wildlife Resources Division (GADNR). Silver Lake WMA was dominated by mature pine forests and forested wetlands. Overstory species were predominately longleaf pine, loblolly pine, slash pine, oaks, and sweetgum (*Liquidambar styraciflua*). Prescribed fire was applied on an approximately 2‐ to 3‐year return interval. For a detailed description of site conditions on Silver Lake WMA, see Wood et al. (2018).

B. F. Grant WMA was owned by the Warnell School of Forestry and Natural Resources at the University of Georgia and was managed jointly by the GADNR and the Warnell School. B. F. Grant was dominated by loblolly pine stands, agricultural lands, mixed hardwood‒pine forests, and hardwood lowlands containing mostly oaks, sweetgum, and hickory (*Carya* spp.). Agricultural lands were mostly grazed mixed fescue (*Festuca* sp.) fields and hay fields planted for rye grass (*Lolium* sp.). Cedar Creek WMA was owned by the U. S. Forest Service (USFS) and managed in partnership with GADNR. Cedar Creek was composed primarily of loblolly pine uplands, mixed hardwood‒pine forests, and hardwood lowlands of similar species composition as B. F. Grant. Prescribed fire was applied on an approximately 3‐ to 5‐year rotation.

In Louisiana, we conducted research on the Kisatchie National Forest (KNF) and Peason Ridge WMA in west‒central Louisiana. The KNF was owned and managed by the USFS, whereas Peason Ridge WMA was jointly owned by the USFS and the US Army. Both areas were composed of pine‐dominated forests consisting of loblolly pine, longleaf pine, slash pine, hardwood riparian zones, and forested wetlands, with forest openings, utility right‐of‐ways, and forest roads distributed throughout. Prescribed fire was applied on an approximately 3‐ to 5‐year return interval. For a detailed description of site conditions on KNF, see Yeldell et al. (2017).

## 3 METHODS

We captured wild turkeys using rocket nets baited with cracked corn during January–March 2014‒2017. We identified sex and determined age of captured individuals based on presence of barring on the ninth and tenth primaries (Pelham & Dickson, 1992). All individuals were given a numbered, riveted aluminum tarsal band and radio‐tagged with a backpack‐style GPS‒VHF transmitter (Guthrie et al., 2011) produced by Biotrack Ltd. We programmed transmitters to take 1 location nightly (23:58:58), and hourly locations between 05:00 and 20:00 until the battery died or the unit were recovered (Cohen, Prebyl, Collier, & Chamberlain, 2018). We released wild turkeys at the capture location immediately following processing. Any individual who died within 3 weeks of release was considered a postrelease mortality and was removed from subsequent analysis.

We monitored live‒dead status daily during the reproductive season using handheld Yagi antennas and R4000 (Advanced Telemetry Systems, Inc.) or Biotracker receivers (Biotrack Ltd.). Live‒dead status was determined via GPS‒VHF transmitter mortality signals scheduled to activate if stationary for 24 hr. We downloaded GPS locations ≥1 per week via a VHF/UHF handheld command unit receiver (Biotrack Ltd.). We viewed GPS locations and determined incubation when female locations became concentrated around a single point for 1–2 days (Collier & Chamberlain, 2011; Conley et al., 2015; Wood et al., 2018; Yeldell et al., 2017). Nesting females were not disturbed or flushed from nest sites during monitoring, but instead were live‒dead checked daily via VHF from a distance of >20 m. All turkey capture, handling, and marking procedures were approved by the Institutional Animal Care and Use Committee at the Louisiana State University Agricultural Center (Protocol No. A2015‐07) and the University of Georgia (Protocol No. A2014 06008Y1A0 and A343701).

Following Yeldell et al. (2017), after nest termination we located nest sites to determine nest fate and confirmed the precise nest location for future analyses. We considered a nest to have been depredated or abandoned if the female left the nest ≤25 days into incubation, or if only intact eggs, no eggs, or egg fragments were found at the nest bowl. After nest termination, we located nests using GPS locations to determine nest fate and to confirm the nest location (Wood et al., 2018; Yeldell et al., 2017).

Females will frequently roost away from the nest site the night before initiating incubation (Conley, Yeldell, Chamberlain, & Collier, 2016), so we censored data from the first day that incubation was confirmed to occur so that we excluded movements potentially associated with laying of the last egg in the clutch from our analysis. Additionally, we censored the day of hatch and the previous day of incubation for successful nests to ensure that movements potentially related to newly hatched broods would not influence inferences related to recess behaviors.

Currently, no published protocol exists for quantifying what constitutes an incubation recess by wild turkeys. Previous work on wild turkeys by Williams et al. (1971) has been widely cited, but inferences from Williams et al. (1971) were based on 10 identified recess events (female leaving and returning to nest) with an average of 1.5 hr per recess. Furthermore, incubating females were irregularly monitored during the incubation period, thus graphical interpolation was used for all individuals observed leaving or returning from recess as described by Williams et al. (1971). Hence, we sought to develop a rigorous, standardized method to identify recess movements using wild turkey spatio‐temporal data. First, based on previous GPS error evaluations (Guthrie et al., 2011), we conservatively buffered each nest site by 27 m (Collier et al., 2019). We then classified any locations >27 m away from the known nest location as a recess movement and considered all locations not at the nest but <27 m from the nest as incubation and not recess movements. We also discarded any GPS fixes that lacked any combination of latitude, longitude, or fix time data. We defined a single recess movement as an individual leaving and then returning to the nest at a later time (e.g., ≥1 location outside of the 27 m buffer). We measured both daily frequency of recess movements, and distance and time of day for each recess movement, and calculated average daily distance traveled on recesses during the entire incubation period for each female. We estimated percentage nest attentiveness (Collier et al., 2019; Skutch, 1962) by removing recesses to obtain the time the female was on the nest (e.g., within the 27 m buffer) and dividing it by the total number of hours spent incubating. As wild turkeys generally have a low probability of nest success (Yeldell et al., 2017), we expected that individuals would be less attentive than other ground‐nesting birds in order to store resources (i.e., bet hedging) for future attempts (Cervencl et al., 2011; Collier et al., 2009; Martin, 1995). Therefore, we then tested for differences between average attentiveness, average daily recess, and average daily distance traveled for successful and unsuccessful nests, using an independent 2‐group *t* test with an *α* = 0.05 in R (R Core Team, 2018). Furthermore, we tested for differences between initial and renesting attempts and average attentiveness, average daily recess, and average daily distance traveled, using an independent 2‐group *t* test with an *α* = 0.05 in R (R Core Team, 2018).

Our nest monitoring data produced a ragged telemetry dataset (Rotella, Dinsmore, & Shaffer, 2004), and we used the nest survival approach outlined by (Dinsmore, White, & Knopf, 2002) to evaluate influences of incubation recess movements on daily nest survival. The ragged telemetry approach serves as a general model for known fate data in program MARK (White & Burnham, 1999) when loss date may not be known exactly and is flexible for integrating time‐dependent individual covariates (Collier et al., 2009; Rotella et al., 2004). For each nesting female, we created an encounter history for the entire incubation period and scaled each nesting event (*k* = 1) to the same start point, as evaluating temporal variation in nest survival was not our objective (Dinsmore et al., 2002). We recorded the last day each nest was known to be alive (*l*) and the final date that the female incubated (*m*) based on our VHF and GPS data (Conley et al., 2015, 2016; Yeldell et al., 2017) and assigned each nest a fate of 0 = survived or 1 = failed. We followed the approach of Collier et al. (2009) and developed time‐dependent covariates for both the daily frequency and distance of recess movements, and time‐dependent covariates for the cumulative values of daily frequency and distance of recess movements. We developed a set of candidate models, which we used to evaluate time‐specific variation in wild turkey behaviors to better understand how variation in behavioral decisions during incubation drive nest survival. Underlying our work was the hypothesis that behavioral changes, manifested via the movement ecology of wild turkeys during nesting, would impact nest success. Our initial expectation was that, generally, increased movements would increase the level of attention on the landscape, which would thus increase nest failure. As such, we included models evaluating fully time‐dependent covariates for daily frequency of recess movements and daily distance of recess movements, as well as cumulative frequency and distance of recess movements (Collier et al., 2009; Franklin, 2001). We also developed time‐specific trend models for cumulative frequency of recesses and distance of recess movements, which assumed that the effect of each covariate did not vary by day and was thus constant over time (Franklin, 2001). We used an information‐theoretic approach (Burnham & Anderson, 2002) to rank candidate models and assess model strength (based on ΔAIC_c_) using the standard from Burnham and Anderson (2002), and estimated daily nest survival for the best fitting candidate model given the data.

We evaluated habitat use by females during the incubation period using dynamic Brownian Bridge movement models (hereafter, dBBMM) to build utilization distributions (UD) at 50% and 99% ranges for each female's incubation range (Byrne, McCoy, Hinton, Chamberlain, & Collier, 2014). Window size and margin size are utilized in a dBBMM to estimate variance across the movement path used to produce a time‒step‐specific UD (Byrne et al., 2014; Kranstauber, Smolla, & Scharf, 2018). We calculated all UDs (Kranstauber et al., 2018) in R (R Core Team, 2018) with R package move (Kranstauber & Smolla, 2013) using a window and margin size equal to 7 (equivalent of 14 hr) and 3 respectively, and a location error of 20 m (Byrne et al., 2014). We kept window and margin size constant to account for changes in GPS sampling frequency because we failed to see any measurable effects of altering these values when we began our analysis (Cohen et al., 2018).

Using 30 m resolution imagery from USGS Landsat‐8 Operational Land Imager, we delineated primary landcover types on our study areas during May for years 2014‒2017, excluding images with ≥10% cloud cover. We chose imagery from May as that was midpoint of the nesting season and assumed landcover types were representative of the entire nesting period. We used an unsupervised classification in ERDAS Image software (Hexagon Geospatial) with 30 classes, and recoded and combined classes to create six unique landcover classes (water, coniferous, deciduous, mixed coniferous‐deciduous, open herbaceous, and human infrastructure). Within each UD, we estimated the proportion of each landcover type to provide an assessment of habitat use by incubating females.

## 4 RESULTS

**Figure 2:**
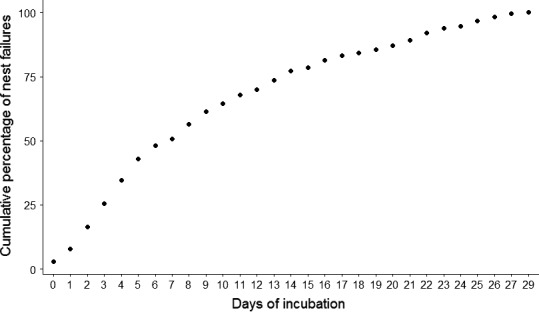
Cumulative nest failure by day of incubation for female Eastern Wild Turkey (*Meleagris gallopavo silvestris*) across multiple study sites in the southeastern United States during 2014–2017

We monitored 332 nesting attempts by 230 (210 adults and 20 juveniles) female wild turkeys during 2014‒2017. We censored 12 nesting attempts because of incomplete GPS data resulting from failed transmitters. Summary metrics for the 320 nesting attempts indicated that females incubated nests an average of 9 days (*SD* = 7.4; median = 7, range = 1‒29), and 75% of nesting attempts failed by day 14 (Figure 2). We removed 51 nesting attempts that were incubated <4 days due to lack of spatial data needed to accurately estimate UDs and used 269 nesting attempts (initial attempts = 189, renesting attempts = 80) by 206 females to quantify recess behaviors and landcover use (Table 1). We identified 9,361 recess movements and 6,529 recess events based on approximately 62,065 hr of incubation across all study sites (Table 2). Based on nesting attempts, mean nest attentiveness was 84.0% (*SD* = 0.13, range = 0%‒98.3%) and did not differ by nest fate (successful mean nest attentiveness = 85.3%, failed mean nest attentiveness = 83.2%; *t* = −1.15, *df* = 115.22, *p* = .253). We found no difference in mean nest attentiveness for successful (84%, *SD* = 14.8) and failed initial nesting attempts (83%, *SD* = 13.7, *t* = −0.46, *df* = 76.21, *p* = .645). However, mean nest attentiveness was greater (*t* = −1.97, *df* = 40.05, *p* = .056) for successful (88%, *SD* = 0.08) than failed renesting attempts (84%, *SD* = 0.09).

**Table 1:** Numbers and percentages of initial nesting attempts, renesting attempts, and fate of 269 nest sites of radio‐tagged female Eastern Wild Turkeys (*Meleagris gallopavo silvestris*) monitored during 2014‒2017

Study site	State	Initial (%)	Renest (%)	Success (%)	Fail (%)
B.F. Grant Wildlife Management Area	Georgia	8 (62)	5 (38)	1 (8)	12 (92)
Cedar Creek Wildlife Management Area	Georgia	24 (77)	7 (23)	5 (16)	26 (84)
Kisatchie National Forest	Louisiana	54 (62)	33 (38)	16 (18)	71 (82)
Peason Ridge Wildlife Management Area	Louisiana	27 (79)	7 (21)	2 (6)	32 (94)
Silver Lake Wildlife Management Area	Georgia	32 (60)	21 (40)	22 (42)	31 (58)
Webb WMA Complex	South Carolina	44 (86)	7 (14)	24 (47)	27 (53)
Total		189 (70)	80 (30)	70 (26)	199 (74)

**Table 2:** Mean and standard deviation (*SD*) of the number of days of incubation, mean (*SD*) number of GPS locations collected while a female was incubating, mean (*SD*) number of recesses per female during incubation, and estimate of nest attentiveness (proportion of time an individual remained on the nest) for 269 nesting attempts made by radio‐tagged female Eastern Wild Turkeys (*Meleagris gallopavo silvestris*) monitored during nesting for each research site across 2014‒2017

Study site	Mean days incubated (*SD*)	Mean no. of GPS locations (*SD*)	Mean recesses per individual (*SD*)	Nest attentiveness, % (*SD*)
B.F. Grant Wildlife Management Area	9 (8.3)	123 (123.0)	24 (24.1)	70 (12.2)
Cedar Creek Wildlife Management Area	15 (8.9)	227 (137.2)	41 (26.3)	73 (8.2)
Kisatchie National Forest	14 (9.0)	201 (144.5)	23 (20.7)	85 (8.1)
Peason Ridge Wildlife Management Area	13 (7.9)	193 (130.0)	17 (17.0)	88 (8.0)
Silver Lake Wildlife Management Area	17 (9.3)	266 (159.1)	20 (14.2)	88 (12.9)
Webb WMA Complex	19 (8.5)	296 (144.6)	26 (17.7)	84 (17.1)
Overall totals/averages	9 (7.4)	231 (149.9)	24.3 (20.6)	84 (12.6)

**Figure 3:**
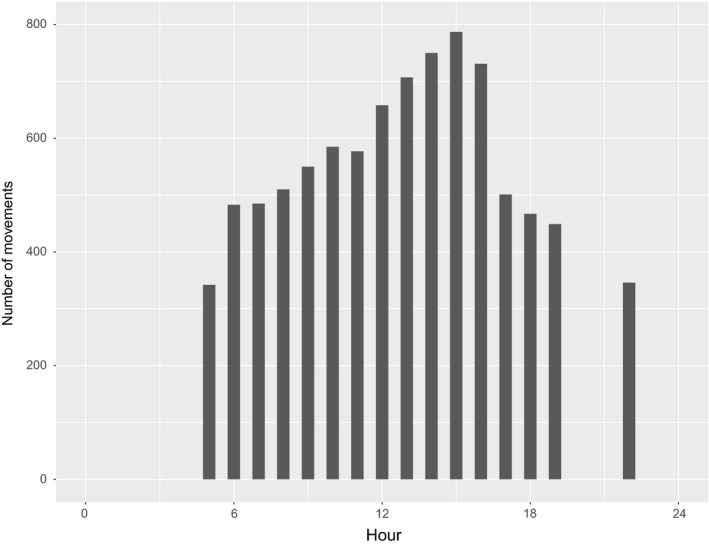
Hourly recess movements for female Eastern Wild Turkey (*Meleagris gallopavo silvestris*) across multiple study sites in the southeastern United States during 2014–2017

We observed that 47% of recess movements occurred between 1,000 and 1,500 (Figure 3), and mean recesses per day was 1.7 (*SD* = 0.79, median = 1.5, range = 0–7). Mean recesses per day did not differ between successful (mean = 1.5, *SD* = 0.78, range 0–7; Table 3) and unsuccessful (mean = 1.7, *SD* = 0.79, range = 0–6) nests (*t* = 1.63, *df* = 121.29, *p* = .106). We found no difference in mean number of daily recesses between initial nesting attempts (mean = 1.7, *SD* = 0.78, range = 0–6) and renesting attempts (mean = 1.6, *SD* = 0.84, range = 0–7, *t* = 0.48, *df* = 138.5, *p* = .631). We also found that the mean number of recesses did not differ between successful (1.6) and unsuccessful (1.7) nests for first nesting attempts (*t* = 0.95, *df* = 86.98, *p* = .345). Additionally, the mean number of recesses did not differ between failed (mean = 1.7, *SD* = 0.81, range = 0–4) and successful renesting attempts (mean = 1.4, *SD* = 0.91, range = 0‒6; *t* = 1.41, *df* = 34.38, *p* = .168).

**Table 3:** Mean and standard deviation (*SD*) of the number of daily incubation recesses for all individual and for individuals with successful, unsuccessful, initial nesting, and renesting attempts for radio‐tagged female Eastern Wild Turkeys (*Meleagris gallopavo silvestris*) monitored during 2014‒2017

Site	Mean number recess (*SD*)	Mean recesses successful nest (*SD*)	Mean recesses failed nest (*SD*)	Mean recesses initial attempt (*SD*)	Mean recesses renest attempt (*SD*)
B.F. Grant Wildlife Management Area	2.5 (0.72)	3	2.4 (0.73)	2.6 (0.56)	2.3 (0.99)
Cedar Creek Wildlife Management Area	2.7 (0.68)	2.5 (0.90)	2.7 (0.65)	2.7 (0.71)	2.7 (0.62)
Kisatchie National Forest	1.7 (0.72)	1.8 (0.93)	1.7 (0.66)	1.7 (0.63)	1.8 (0.84)
Peason Ridge Wildlife Management Area	1.3 (0.68)	1.6 (0.92)	1.2 (0.68)	1.3 (0.71)	1.1 (0.61)
Silver Lake Wildlife Management Area	1.3 (0.44)	1.3 (0.51)	1.2 (0.39)	1.3 (0.45)	1.2 (0.44)
Webb WMA Complex	1.5 (0.65)	1.3 (0.65)	1.6 (0.63)	1.5 (0.64)	1.1 (0.60)
Overall totals/averages	1.7 (0.79)	1.5 (0.78)	1.7 (0.79)	1.7 (0.78)	1.6 (0.84)

Average daily distance moved during incubation was 84.8 m (*SD* = 30.9, range = 0–998.5 m). Females with failed nesting attempts did not move farther on average each day (87.2 m, *SD* = 34.3, range = 0–998.5 m) than successful females (84.7 m, *SD* = 32.3, range = 0–998.5 m; *t* = −0.289, *df* = 54.49, *p* = .773). Average daily distance moved did not differ between initial (mean = 82.9 m, *SD* = 31.5, range = 0–998.5 m) and renesting attempts (mean = 86.5 m, *SD* = 27.9, range = 0–724.5 m; *t* = −0.455, *df* = 53.89, *p* = .651). We found no difference in average daily distance moved during incubation for successful first nesting attempts (83.8 m, *SD* = 33.2, range = 0–998.5 m) and failed first nesting attempts (92.2 m, *SD* = 67.0, range = 0–780.7 m, *t* = −0.601, *df* = 39.19, *p* = .551). Average daily distance moved was similar (*t* = −1.105, *df* = 49.32, *p* = .274) for failed renesting attempts (91.07 m, *SD* = 35.6, range = 0‒724.48 m) relative to successful attempts (81.4 m, *SD* = 28.0, range = 0‒522.2 m).

**Figure 4:**
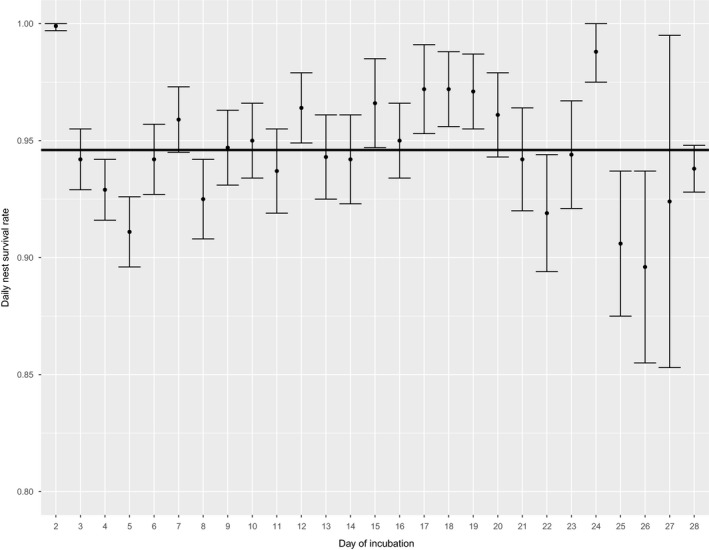
Estimated daily nest survival for Eastern Wild Turkey (*Meleagris gallopavo silvestris*) nests across the incubation period based on the mean cumulative daily distance moved during recesses across multiple study sites in the southeastern United States during 2014–2017. Solid line shows the estimated mean daily nest survival

The best model for estimating influences of incubation behaviors on daily nest survival included cumulative recess movements made by an incubating female as a covariate that was time‐dependent (Table 4), and there was little model selection uncertainty (*w_i_* = 0.97) in our candidate model set. Based on the best fitting model, daily nest survival varied over the 28‐day period depending on the estimated cumulative daily distances moved (Table 5, Figure 4, Appendix S1). We note that the estimated overall nest survival rate under our best fitting model, when estimated at the mean values for cumulative daily distance moved, was 0.22 (CI = 0.16‒0.28), which was comparable to a naïve estimate of nest success (24%) from our data.

**Table 4:** Candidate models used to examine the effect of frequency of daily recess movements away from the nest, mean distance of daily recess movements away from the nest, mean cumulative daily distance, which is the total distance throughout the incubation period, mean cumlative recess movements total number of recesses througout the incubation period, and time on daily nest survival (DSR) of radio‐tagged Eastern Wild Turkeys (*Meleagris gallopavo silvestris*) monitored in the southeastern United States 2014‒2017

Model notation	*k*	Deviance	ΔAIC_c_	*w_i_*
DSR (Cumulative daily distance moved by day)^a^	27	1506.45	0	0.977
DSR (Cumulative recesses)^a^	27	1515.56	9.10	0.103
DSR (Daily distance moved)^a^	27	1516.33	9.87	0.007
DSR (Constant days 1–11, Cumulative distance moved days 12–21, Constant days 22–28)	13	1546.50	11.76	<0.001
DSR (Daily number of recesses)^a^	27	1518.39	11.94	<0.001
DSR (Number of daily recesses + daily distance moved + Number of daily recesses*daily distance moved)^a^	4	1569.28	16.45	<0.001
DSR (Cumulative daily distance moved)^b^	2	1583.31	26.48	<0.001
DSR (Daily distance moved)^b^	2	1583.66	26.82	<0.001
DSR (Cumulative recesses)^b^	2	1583.66	26.83	<0.001
DSR (Constant)	1	1585.78	26.94	<0.001
DSR (Daily number of recesses)^b^	2	1584.57	27.73	<0.001

a Effect is fully time dependent.

b Effect is a linear time dependent trend.

**Table 5:** Mean cumulative distance moved (*m*) and associated predicted daily survival rate (DSR (*SE*)) estimated at the mean cumulative daily distance moved for each day of the incubation period

Day of incubation	Mean cumulative distance moved (m)	DSR	*SE*	Range (m)
2	125	0.999	0.002	0–724
3	225	0.942	0.013	0–1180
4	313	0.929	0.013	29–1,775
5	396	0.911	0.015	32–2,365
6	461	0.942	0.015	32–1,645
7	539	0.959	0.014	61–1,850
8	612	0.925	0.017	107–1,880
9	694	0.947	0.016	107–1,917
10	782	0.950	0.016	165–2,069
11	861	0.937	0.018	165–2,204
12	944	0.964	0.015	165–2,490
13	1,019	0.943	0.018	230–2,569
14	1,087	0.942	0.019	253–2,569
15	1,176	0.966	0.019	253–2,681
16	1,275	0.950	0.016	282–2,681
17	1,369	0.972	0.019	409–2,858
18	1,465	0.972	0.016	483–3,004
19	1,557	0.971	0.016	541–3,374
20	1,634	0.961	0.018	541–4,018
21	1,704	0.942	0.022	541–4,092
22	1,826	0.919	0.025	623–4,417
23	1,917	0.944	0.023	690–4,456
24	2,014	0.988	0.013	761–4,004
25	2,105	0.906	0.031	770–4,215
26	2,220	0.896	0.041	820–4,412
27	2,438	0.924	0.071	840–4,498
28	2,911	0.938	0.010	2,190–4,336
29	3,114	—	—	2,317–3,642
30	3,642	—	—	3,642

Note

Additionally, we have provided the associated range of cumulative distance (*m*) moved during incubation for radio‐tagged Eastern Wild Turkey (*Meleagris gallopavo silvestris*) monitored across all study sites during 2014–2017, which can be combined with the *β* estimates (Appendix S1) to develop incubation day specific estimates of daily nest survival across the entire incubation period and range of estimates cumulative movements.

Mean 50% UD range size during incubation was 0.2 ha (*SD* = 0.2, range = 0.03‒2.09 ha), whereas 99% UDs averaged 11.4 ha (*SD* = 17.0, range = 0.23‒111.0 ha). Pine was the dominant habitat type (63%; *SD* = 30.2, range = 0%‒100%, Table 6) within incubation ranges across all study sites. We found that failed nests (64% pine) were associated with greater percentages of pine than successful nests (56%) in the 99% UD (*t* = 1.981, *df* = 114.73, *p* = .050; Table 7) Furthermore, greater percentages of pine within the 50% UD were associated with reduced nest success (successful nest attempts pine percentage = 49%, unsuccessful nest attempts percentage of pine = 69%; *t* = 3.526, *df* = 109.84, *p* < .001).

**Table 6:** Mean size of 50% and 99% utilization distributions for incubation recess ranges, and proportion of habitat types within the ranges for female Eastern Wild Turkeys (*Meleagris gallopavo silvestris*) during 2014‒2017

Site	Mean incubation range (ha)	Mean proportion pine 99% (*SD*)	Mean proportion hardwoods 99% (*SD*)	Mean proportion mixed 99% (*SD*)	Mean proportion open 99% (*SD*)	Mean proportion human infrastructure 99% (*SD*)	Mean proportion water 99% (*SD*)	Mean proportion pine 50% (*SD*)	Mean proportion hardwoods 50% (*SD*)	Mean proportion mixed 50% (*SD*)	Mean proportion open 50% (*SD*)	Mean proportion human infrastructure 50% (*SD*)	Mean proportion water 50% (*SD*)
B.F. Grant Wildlife Management Area	27.6 (27.2)	77.3 (18.5)	1.0 (1.9)	11.0 (10.8)	9.8 (11.4)	0 (0)	0.1 (1.5)	82.3 (34.2)	0 (0)	11.4 (27.9)	11.7 (29.7)	0 (0)	0 (0)
Cedar Creek Wildlife Management Area	29.2 (28.8)	75.7 (23.7)	1.9 (6.4)	11.9 (16.9)	9.2 (14.1)	0 (0)	1.4 (5.3)	82.9 (32.6)	2.6 (14.3)	3.2 (10.4)	10.6 (24.9)	0 (0)	0.6 (3.7)
Kisatchie National Forest	9.1 (11.8)	73.5 (26.0)	3.3 (7.4)	9.4 (18.3)	8.8 (18.0)	3.2 (6.8)	1.8 (8.3)	75.7 (35.7)	4.2 (16.4)	9.1 (24.0)	7.7 (22.2)	1.7 (8.3)	1.6 (11.1)
Peason Ridge Wildlife Management Area	6.9 (7.7)	54.7 (24.1)	1.8 (3.8)	22.0 (21.1)	12.7 (19.7)	8.5 (16.9)	0 (2.0)	65.1 (39.9)	0 (0)	21.0 (34.0)	14.3 (31.0)	5.8 (16.8)	0 (0)
Silver Lake Wildlife Management Area	5.5 (5.6)	65.8 (26.0)	11.3 (13.5)	3.7 (12.0)	14.9 (22.0)	1.2 (2.5)	3.0 (11.0)	65.0 (42.0)	9.6 (24.0)	3.7 (18.0)	19.8 (36.4)	1.4 (10.2)	0.3 (1.2)
Webb WMA Complex	9.5 (15.2)	33.4 (29.7)	7.9 (10.2)	41.9 (36.3)	5.0 (12.1)	0.6 (1.7)	11.1 (15.0)	33.4 (40.1)	1.8 (6.7)	47.4 (47.2)	3.9 (15.6)	0 (0)	13.4 (29.1)

**Table 7:** Mean proportion of habitat types within the 50% and 99% ranges for female Eastern Wild Turkeys (*Meleagris gallopavo silvestris*) based on nest fate (successful or fail), nesting attempt (initial or renest), and age (adult or juvenile) during 2014‒2017

	Mean proportion pine 99% (*SD*)	Mean proportion hardwoods 99% (*SD*)	Mean proportion mixed 99% (*SD*)	Mean proportion open 99% (*SD*)	Mean proportion human infrastructure 99% (*SD*)	Mean proportion water 99% (*SD*)	Mean proportion pine 50% (*SD*)	Mean proportion hardwoods 50% (*SD*)	Mean proportion mixed 50% (*SD*)	Mean proportion open 50% (*SD*)	Mean proportion human infrastructure 50% (*SD*)	Mean proportion water 50% (*SD*)
Successful	0.50 (0.31)	0.10 (0.16)	0.19 (0.31)	0.10 (0.20)	0.02 (0.08)	0.04 (0.09)	0.50 (0.43)	0.10 (0.28)	0.19 (0.36)	0.14 (0.31)	0.01 (0.09)	0.06 (0.14)
Fail	0.59 (0.33)	0.08 (0.13)	0.18 (0.24)	0.10 (0.18)	0.02 (0.04)	0.04 (0.11)	0.59 (0.43)	0.08 (0.20)	0.17 (0.32)	0.12 (0.27)	0.01 (0.08)	0.03 (0.21)
Initial attempt	0.56 (0.33)	0.08 (0.13)	0.19 (0.27)	0.10 (0.17)	0.02 (0.08)	0.04 (0.11)	0.57 (0.43)	0.08 (0.22)	0.20 (0.35)	0.10 (0.26)	0.01 (0.06)	0.04 (0.17)
Renest attempt	0.59 (0.33)	0.09 (0.16)	0.12 (0.24)	0.15 (0.21)	0.02 (0.05)	0.03 (0.11)	0.59 (0.44)	0.09 (0.23)	0.11 (0.28)	0.16 (0.32)	0.02 (0.12)	0.03 (0.12)
Adult	0.58 (0.33)	0.08 (0.19)	0.18 (0.26)	0.10 (0.18)	0.02 (0.07)	0.04 (0.11)	0.59 (0.44)	0.08 (0.23)	0.17 (0.33)	0.12 (0.28)	0.02 (0.09)	0.03 (0.15)
Juvenile	0.51 (0.34)	0.08 (0.16)	0.23 (0.24)	0.11 (0.22)	0.01 (0.04)	0.06 (0.11)	0.46 (0.41)	0.07 (0.19)	0.25 (0.33)	0.13 (0.31)	0 (0)	0.09 (0.25)

## 5 DISCUSSION

Our results indicated that the average number of days a female wild turkey incubated a nest was 9 days, and 75% of nesting attempts failed by day 14. Recess movements were distributed throughout the day, with most occurring between 1,000 and 1,500, which varied from previous works with wild turkeys where recesses were observed during early morning and late afternoon hours (Green, 1982; Spohr, 2001; Williams et al., 1971). We observed that on average, female wild turkeys made 1–2 recesses per day. Nest attentiveness estimates were lower (84%) relative to other ground‐nesting galliforms, including Greater Sage‐Grouse (96%; *Centrocercus urophasianus*), Greater Prairie‐Chicken (95% *Tympanuchus cupido*), and White‐tailed Ptarmigan (95%; *Lagopus leucura*; Wiebe & Martin, 2000, Deeming, 2002, Coates & Delehanty, 2008, Winder et al., 2016). However, our estimates of nest attentiveness were similar to other related species such as the Sichuan Partridge (82%, *Arborophila rufipectus*) and Blood Pheasant (72%, *Ithaginis cruentus*; Fu et al., 2017; Jia et al., 2010).

Our survival analysis indicated that variation in daily nest survival of female wild turkeys was best described based on estimates of cumulative daily distance moved during recesses. Interestingly, our results indicated that daily nest survival was generally positively impacted by cumulative daily movements by females, except early and late in the incubation period. Exposure of wild turkeys to potential causes of mortality is highest during the reproductive season (Palmer, Hurst, Stys, Smith, & Burk, 1993), and exposure of nests is usually tied to behavioral activities undertaken by females (Collier et al., 2009). Our results suggest that distances moved away from nests may confer positive benefits to daily nest survival, especially during the middle of the incubation period (see *β* estimates, Appendix S1). However, as the incubation period nears completion, increased movements tend to lead to lower nest survival, especially in the last 4–5 days of incubation. Time spent away from nests by Greater Sage‐Grouse declined as predator abundance increased, likely driven by the need to reduce interactions with nest predators (Coates & Delehanty, 2008; Dudko et al., 2019). For wild turkeys in our study, decreased movements during the beginning and end of the incubation period may be associated with level of predation risk, and the trade‐off between nest success and individual survival. Wild turkeys are thought to maximize individual survival relative to nest survival (Collier et al., 2009), so in systems with increased predation risk, we would expect that individuals choosing to maximize survival over reproduction would make movements farther from nest sites during incubation (Behrens, Ruff, Harms, & Dinsmore, 2019; Cervencl et al., 2011; Smith et al., 2012). We speculate that as nests near the expected hatch dates, perhaps, females are more likely to prioritize reproductive success over individual survival.

Our results indicated that wild turkeys made relatively few recesses (1–2 recesses per day) compared to other species, but we did not find evidence that nest survival was associated with number of daily recess movements. Works on fitness consequences of recess movements have been noted in White‐tailed Ptarmigan, wherein individuals making fewer and shorter duration movements have a decreased probability of nest predation and increased nest survival (Wiebe & Martin, 1997). Similarly, shorebird species (Red Phalaropes, *Phalaropus fulicarius*, Little Stints, *Calidris minuta*) that made fewer recesses during incubation had a greater chance of nest success (Smith et al., 2012). Reductions in nest attentiveness during incubation have been found to increase as the nesting period continues (Kessler, 1962) and are thought to change based on overall body condition of the individual based on available resources within the surrounding landscape (Aldrich & Raveling, 1983). We found that increased levels of pine landcover type within the incubation ranges had negative impacts on nest success. We suspect that the observed relationships between pine landcover within incubation ranges and nest success stemmed from the relatively coarse resolution of our assessment and overall availability of the pine landcover type ($\bar{x}$ = 63%) as resource selection during nesting is assumed to be adaptive and has fitness consequences. For example, White‐Rumped Sandpipers (*Calidris fuscicollis*) habitat selection drove nest success due to variation in food abundance (Smith, Gilchrist, & Smith, 2007). Conversely, Martin (1993) suggested that avian species choose nest sites not based upon food availability but to reduce risk of predation. Thus, we suggest further behavioral evaluations within finer landcover types may indicate either limitation in resources, increased predation risk, or potential movement restrictions, which may influence nest success or failure.

Our collective understanding of incubation behaviors in wild turkeys is based on limited literature (Green, 1982; Spohr, 2001; Williams et al., 1971). Our approach to assessing recess behavior was the first to describe a standard, repeatable approach for identifying recess frequency and movements. We found that throughout incubation, individual females varied in regard to numbers of daily recesses taken, which contradicts previous works suggesting that females recess only once per day or even once every several days (Williams et al., 1971). Collectively, our results suggest that recess behaviors by wild turkeys are more nuanced than previously believed and may have important implications to fitness. We offer that behavioral decisions made during incubation may be a more influential driver of nest survival than previously expected (Conley et al., 2015). The ultimate drivers of behavioral decisions during incubation are unknown, but are likely made based upon a suite of abiotic and biotic factors such as body condition, ambient temperature/precipitation, resource availability, and vegetative conditions (Aldrich & Raveling, 1983; Conway & Martin, 2000; Deeming, 2002; Smith et al., 2012; Webb, 1987). Furthermore, individual behaviors/time allocation patterns can be altered by predation risk (Laundré, Hernández, & Ripple, 2010), and as such, the intensity of nest predation likely underlies plasticity in behavioral responses or antipredator strategies (Conway & Martin, 2000; Ghalambor & Martin, 2002; Martin, 2002). We predict that individual incubation behaviors may be influenced by vegetation composition and predation risk. Therefore, exploration of the potential that female turkeys use variable strategies to improve reproductive fitness should be evaluated in future research.

## CONFLICT OF INTEREST

None declared.

## AUTHORS CONTRIBUTION

NWB, MJC, and BAC conceived the project. All authors contributed to field experimental design development, data collection, statistical analysis and manuscript development, and review and gave final approval for publication.

## Data Availability

All raw survival data from this study are available upon request or can be accessed on Dryad (https://doi.org/10.5061/dryad.2547d7wmn).

## References

[ece35843-bib-0001] Aldrich, T. W. , & Raveling, D. G. (1983). Effects of experience and body weight on incubation behavior of Canada Geese. The Auk, 100, 670–679. 10.1093/auk/100.3.670

[ece35843-bib-0002] Badyaev, A. V. , Etges, W. J. , & Martin, T. E. (1996). Habitat sampling and habitat selection by female Wild Turkeys: Ecological correlates and reproductive consequences. The Auk, 113, 636–646. 10.2307/4088984

[ece35843-bib-0003] Batary, P. , & Baldi, A. (2004). Evidence of an edge effect on avian nest success. Conservation Biology, 18, 389–400. 10.1111/j.1523-1739.2004.00184.x

[ece35843-bib-0004] Behrens, C. , Ruff, Z. J. , Harms, T. M. , & Dinsmore, S. J. (2019). Predator density influences nest attendance of Yellow‐Headed Blackbirds *Xanthocephalus xanthocephalus* . Ibis, 161, 679–685. 10.1111/ibi.12705

[ece35843-bib-0005] Burnam, J. S. , Turner, G. , Ellis‐Felege, S. N. , Palmer, W. E. , Sisson, D. C. , & Carroll, J. P. (2012). Patterns of incubation behavior in northern bobwhites In Pietz P. J., Ribic C. A., & Thompson F. R. III (Eds.), Video surveillance of nesting birds (pp. 77–88). Berkeley, CA: University of California Press.

[ece35843-bib-0006] Burnham, K. P. , & Anderson, D. R. (2002). Model selection and multimodel inference: A practical information theoretic approach (2nd ed.). New York, NY: Springer‐Verlag.

[ece35843-bib-0007] Byrne, M. E. , & Chamberlain, M. J. (2013). Nesting ecology of Wild Turkeys in a bottomland hardwood Forest. The American Midland Naturalist, 170, 95–110. 10.1674/0003-0031-170.1.95

[ece35843-bib-0008] Byrne, M. E. , McCoy, J. C. , Hinton, J. W. , Chamberlain, M. J. , & Collier, B. A. (2014). Using dynamic Brownian bridge movement modelling to measure temporal patterns of habitat selection. Journal of Animal Ecology, 83, 1234–1243. 10.1111/1365-2656.12205 24460723

[ece35843-bib-0009] Cervencl, A. , Esser, W. , Maier, M. , Oberdiek, N. , Thyen, S. , Wellbrock, A. , & Exo, K. M. (2011). Can differences in incubation patterns of Common Redshanks *Tringa totanus* be explained by variations in predation risk? Journal of Ornithology, 152, 1033–1043. 10.1007/s10336-011-0696-z

[ece35843-bib-0010] Chamberlain, M. J. , & Leopold, B. D. (2000). Habitat sampling and selection by female Wild Turkeys during preincubation. Wilson Bulletin, 112, 326–331. 10.1676/0043-5643(2000)112[0326:HSASBF]2.0.CO;2

[ece35843-bib-0011] Coates, P. S. , & Delehanty, D. J. (2008). Effects of environmental factors on incubation patterns of Greater Sage‐Grouse. Condor, 110, 627–638. 10.1525/cond.2008.8579

[ece35843-bib-0012] Cohen, B. S. , Prebyl, T. J. , Collier, B. A. , & Chamberlain, M. J. (2018). Home range estimator method and GPS sampling schedule affect habitat selection inferences for Wild Turkeys. Wildlife Society Bulletin, 42, 150–159. 10.1002/wsb.845

[ece35843-bib-0013] Collier, B. A. , & Chamberlain, M. J. (2011). Redirecting research for Wild Turkeys using global positioning system transmitters In Proceedings of the National Wild Turkey Symposium (vol. 10, pp. 81–92).

[ece35843-bib-0014] Collier, B. A. , Fyffe, N. , Smallwood, A. , Oleson, B. , Bakner, N. W. , Heffelfinger, J. R. , & Chamberlain, M. J. (2019). Reproductive ecology of Goulds Wild Turkeys in Arizona. Wilson Journal of Ornithology, 131, 667–679.

[ece35843-bib-0015] Collier, B. A. , Melton, K. B. , Hardin, J. B. , Silvy, N. J. , & Peterson, M. J. (2009). Impact of reproductive effort on survival of Rio Grande Wild Turkey *Meleagris gallopavo intermedia* hens in Texas. Wildlife Biology, 15, 370–379.

[ece35843-bib-0016] Conley, M. D. , Oetgen, J. G. , Barrow, J. , Chamberlain, M. J. , Skow, K. L. , & Collier, B. A. (2015). Habitat selection, incubation, and incubation recess ranges of nesting female Rio Grande Wild Turkeys in Texas In Proceedings of the National Wild Turkey Symposium (vol. 11, pp. 117–126).

[ece35843-bib-0017] Conley, M. D. , Yeldell, N. A. , Chamberlain, M. J. , & Collier, B. A. (2016). Do movement behaviors identify reproductive habitat sampling for Wild Turkeys? Ecology and Evolution, 6, 7103–7112. 10.1002/ece3.2401 28725385 PMC5513226

[ece35843-bib-0018] Conway, C. J. , & Martin, T. E. (2000). Evolution of passerine incubation behavior: Influence of food, temperature, and nest predation. Evolution, 54, 670–685. 10.1111/j.0014-3820.2000.tb00068.x 10937242

[ece35843-bib-0019] Deeming, D. C. (2002). Avian incubation: Behavior, environment and evolution. New York, NY: Oxford University Press.

[ece35843-bib-0020] Deeming, D. C. , & Reynolds, S. J. (2015). Nests, eggs, and incubation: New ideas about avian reproduction. Oxford, UK: Oxford University Press.

[ece35843-bib-0022] Dinsmore, S. J. , White, G. C. , & Knopf, F. L. (2002). Advanced techniques for modeling avian nest survival. Ecology, 83, 3476–3488. 10.1890/0012-9658(2002)083[3476:ATFMAN]2.0.CO;2

[ece35843-bib-0024] Dudko, J. E. , Coates, P. S. , & Delehanty, D. J. (2019). Movements of female Sage Grouse *Centrocercus urophasianus* during incubation recess. Ibis, 161, 222–229.

[ece35843-bib-0025] Fontaine, J. , & Martin, T. (2006). Parent birds assess nest predation risk and adjust their reproductive strategies. Ecology Letters, 9, 428–434. 10.1111/j.1461-0248.2006.00892.x 16623728

[ece35843-bib-0026] Franklin, A. B. (2001). Exploring ecological relationships in survival and estimating rates of population change using program MARK In Field R., Warren R. J., Okarma H., & Sievert P. R. (Eds.), Wildlife, land, and people: Priorities for the 21st century (pp. 290‒296). Bethesda, MD: The Wildlife Society.

[ece35843-bib-0027] Fu, Y. , Dai, B. , Wen, L. , Chen, B. , Dowell, S. , & Zhang, Z. (2017). Unusual incubation behavior and embryonic tolerance of hypothermia in the Sichuan Partridge (*Arborophila rufipectus*). Journal of Ornithology, 158, 707–715. 10.1007/s10336-016-1422-7

[ece35843-bib-0028] Ghalambor, C. K. , & Martin, T. E. (2002). Comparative manipulation of predation risk in incubating birds reveals variability in the plasticity of responses. Behavioral Ecology, 13, 101–108. 10.1093/beheco/13.1.101

[ece35843-bib-0029] Green, H. E. (1982). Reproductive behavior of female Wild Turkeys in northern Lower Michigan. Journal of Wildlife Management, 46, 1065–1071. 10.2307/3808242

[ece35843-bib-0030] Guthrie, J. D. , Byrne, M. E. , Hardin, J. B. , Kochanny, C. O. , Skow, K. L. , Snelgrove, R. T. , … Collier, B. A. (2011). Evaluation of a Global Positioning System backpack transmitter for Wild Turkey research. Journal of Wildlife Management, 75, 539–547. 10.1002/jwmg.137

[ece35843-bib-0031] Healy, W. M. (1992). Behavior In Dickson J. G. (Ed.), The Wild Turkey: Biology and management (pp. 46–65). Mechanicsburg, PA: Stackpole.

[ece35843-bib-0032] Jia, C.‐X. , Sun, Y.‐H. , & Swenson, J. E. (2010). Unusual incubation behavior and embryonic tolerance of hypothermia by the Blood Pheasant (*Ithaginis cruentus*). The Auk, 127, 926–931.

[ece35843-bib-0033] Jones, J. (2001). Habitat selection studies in avian ecology: A critical review. The Auk, 118, 557–562. 10.1093/auk/118.2.557

[ece35843-bib-0034] Kessler, F. (1962). Measurement of nest attentiveness in the Ring‐Necked Pheasant. The Auk, 79, 702–705. 10.2307/4082649

[ece35843-bib-0035] Kranstauber, B. , & Smolla, M. (2013). move: Visualizing and analyzing animal track data. Free R software package.

[ece35843-bib-0036] Kranstauber, B. , Smolla, M. , & Scharf, A. K. (2018). move: Visualizing and analyzing animal track data. R package version 3.1.0.

[ece35843-bib-0037] Laundré, J. W. , Hernández, L. , & Ripple, W. J. (2010). The landscape of fear: Ecological implications of being afraid. Open Ecology Journal, 3, 1–7.

[ece35843-bib-0038] Little, A. R. , Chamberlain, M. J. , Conner, L. M. , & Warren, R. J. (2016). Habitat selection of Wild Turkeys in burned longleaf pine savannas. Journal of Wildlife Management, 80, 1280–1289. 10.1002/jwmg.21114

[ece35843-bib-0039] Martin, J. , Juhan, S., Jr. , Palmer, W. , & Carroll, J. (2015). Incubation and predation ecology of Wild Turkey nests: A cautionary case study regarding video camera surveillance In Proceedings of the National Wild Turkey Symposium (vol. 11, pp. 295–301).

[ece35843-bib-0040] Martin, T. E. (1993). Nest predation and nest sites. BioScience, 43, 523–532. 10.2307/1311947

[ece35843-bib-0041] Martin, T. E. (1995). Avian life history evolution in relation to nest sites, nest predation, and food. Ecological Monographs, 65, 101–127. 10.2307/2937160

[ece35843-bib-0042] Martin, T. E. (2002). A new view of avian life‐history evolution tested on an incubation paradox. Proceedings of the Royal Society of London. Series B: Biological Sciences, 269, 309–316. 10.1098/rspb.2001.1879 11839200 PMC1690888

[ece35843-bib-0043] Naylor, B. J. , Szuba, K. J. , & Bendell, J. F. (1988). Nest cooling and recess length of incubating Spruce Grouse. Condor, 90, 489–492. 10.2307/1368580

[ece35843-bib-0044] Orians, G. H. , & Wittenberger, J. F. (1991). Spatial and temporal scales in habitat selection. The American Naturalist, 137, S29–S49. 10.1086/285138

[ece35843-bib-0045] Palmer, W. E. , Hurst, G. A. , Stys, J. E. , Smith, D. R. , & Burk, J. D. (1993). Survival rates of wild turkey hens in loblolly pine plantations in Mississippi. The Journal of Wildlife Management, 57, 783–789. 10.2307/3809080

[ece35843-bib-0046] Pelham, P. H. , & Dickson, J. G. (1992). Physical characteristics In Dickson J. G. (Ed.), The Wild Turkey: Biology and management (pp. 32–45). Mechanicsburg, PA: Stackpole.

[ece35843-bib-0047] R Core Team (2018). R: A language and environment for statistical computing. Vienna, Austria: R Foundation for Statistical Computing Retrieved from http://www.R-project.org

[ece35843-bib-0048] Rotella, J. J. , Dinsmore, S. J. , & Shaffer, T. L. (2004). Modeling nest‐survival data: A comparison of recently developed methods that can be implemented in MARK and SAS. Animal Biodiversity and Conservation, 27, 187–205.

[ece35843-bib-0049] Skutch, A. F. (1962). The constancy of incubation. Wilson Bulletin, 74, 115–152.

[ece35843-bib-0050] Smith, P. A. , Gilchrist, H. G. , & Smith, J. N. M. (2007). Effects of nest habitat, food, and parental behavior on shorebird nest success. Condor, 109, 15–31. 10.1093/condor/109.1.15

[ece35843-bib-0051] Smith, P. A. , Tulp, I. , Schekkerman, H. , Gilchrist, H. G. , & Forbes, M. R. (2012). Shorebird incubation behaviour and its influence on the risk of nest predation. Animal Behaviour, 84, 835–842. 10.1016/j.anbehav.2012.07.004

[ece35843-bib-0052] Spohr, S. M. (2001). Variables influencing nest success of eastern wild turkeys in Connecticut: Nesting habitat, home range‐scale fragmentation, and nest attentiveness. Thesis. University of Maine, Orono, ME.

[ece35843-bib-0053] Streich, M. M. , Little, A. R. , Chamberlain, M. J. , Conner, L. M. , & Warren, R. J. (2015). Habitat characteristics of Eastern Wild Turkey nest and ground‐roost sites in 2 longleaf pine forests. Journal of the Southeastern Association of Fish and Wildlife Agencies, 2, 164–170.

[ece35843-bib-0054] Webb, D. (1987). Thermal tolerance of avian embryos: A review. Condor, 89, 874–898. 10.2307/1368537

[ece35843-bib-0055] White, G. C. , & Burnham, K. P. (1999). Program MARK: Survival estimation from populations of marked animals. Bird Study, 46, S120–S139. 10.1080/00063659909477239

[ece35843-bib-0056] Wiebe, K. L. , & Martin, K. (1997). Effects of predation, body condition and temperature on incubation rhythms of White‐tailed Ptarmigan *Lagopus leucurus* . Wildlife Biology, 3, 219–227.

[ece35843-bib-0057] Wiebe, K. L. , & Martin, K. (2000). The use of incubation behavior to adjust avian reproductive costs after egg laying. Behavioral Ecology and Sociobiology, 48, 463–470. 10.1007/s002650000259

[ece35843-bib-0058] Wightman, P. H. , Cantrell, J. R. , Ruth, C. R. , Byrne, M. E. , Chamberlain, M. J. , & Collier, B. A. (2018). Impact of supplemental feeding for Northern Bobwhite on movement ecology of Eastern wild turkeys in South Carolina. Journal of the Southeastern Association of Fish and Wildlife Agencies, 5, 114–124.

[ece35843-bib-0059] Williams, L. E., Jr. , Austin, D. H. , Peoples, T. E. , & Phillips, R. W. (1971). Laying data and nesting behavior of wild turkeys. Proceedings of the Southeastern Association of Game and Fish Commissioners, 25, 90–106.

[ece35843-bib-0060] Winder, V. L. , Herse, M. R. , Hunt, L. M. , Gregory, A. J. , McNew, L. B. , & Sandercock, B. K. (2016). Patterns of nest attendance by female greater prairie‐chickens (*Tympanuchus cupido*) in northcentral Kansas. Journal of Ornithology, 157, 733–745. 10.1007/s10336-016-1330-x

[ece35843-bib-0061] Wood, J. D. , Cohen, B. S. , Prebyl, T. J. , Conner, L. M. , Collier, B. A. , & Chamberlain, M. J. (2018). Time‐since‐fire and stand seral stage affect habitat selection of eastern wild turkeys in a managed longleaf pine ecosystem. Forest Ecology and Management, 411, 203–212. 10.1016/j.foreco.2018.01.033

[ece35843-bib-0062] Yeldell, N. A. , Cohen, B. S. , Little, A. R. , Collier, B. A. , & Chamberlain, M. J. (2017). Nest site selection and nest survival of eastern wild turkeys in a pyric landscape. Journal of Wildlife Management, 81, 1073–1083. 10.1002/jwmg.21267

