# Supplementary material for: Incubation recess behaviors influence nest survival of Wild Turkeys
Source: Ecol Evol. 2019 Nov 21;9(24):14053–65. doi: 10.1002/ece3.5843 (PMC6953688; doi:10.1002/ece3.5843)
Supplement: Supplementary file 1 [file ECE3-9-14053-s001.docx]

**Appendix**

| Appendix S1. Beta estimates, standard error, and confidence intervals for daily survival rate estimation (DSR) for Eastern Wild Turkeys (*Meleagris gallopavo silvestris*) nests across all study sites during years 2014–2017. | | | |
| --- | --- | --- | --- |
| Day of Incubation | Beta | Standard Error | Confidence Intervals |
| Intercept | 2.72571 | 0.16742 | 2.38756-3.04386 |
| 1 | 0.00000 | 0.00000 | 0.00000-0.00000 |
| 2 | 0.01801 | 0.00791 | 0.00250-0.03352 |
| 3 | 0.00023 | 0.00081 | -0.00136-0.00182 |
| 4 | -0.00035 | 0.00056 | -0.00144-0.00073 |
| 5 | -0.00085 | 0.00046 | -0.00175-0.00005 |
| 6 | 0.00014 | 0.00056 | -0.00095-0.00123 |
| 7 | 0.00070 | 0.00062 | -0.00051-0.00191 |
| 8 | -0.00029 | 0.000039 | -0.00106-0.00048 |
| 9 | 0.00021 | 0.00044 | -0.00066-0.00107 |
| 10 | 0.00027 | 0.00043 | -0.00057-0.00110 |
| 11 | -0.00019 | 0.00035 | -0.00071-0.00067 |
| 12 | 0.00056 | 0.00046 | -0.00034-0.00145 |
| 13 | 0.00009 | 0.00034 | -0.00058-0.00075 |
| 14 | 0.00006 | 0.00032 | -0.00056-0.00068 |
| 15 | 0.00050 | 0.00041 | -0.00029-0.00130 |
| 16 | 0.00016 | 0.00031 | -0.00044-0.00077 |
| 17 | 0.00057 | 0.00041 | -0.00023-0.00138 |
| 18 | 0.00052 | 0.00038 | -0.00023-0.00128 |
| 19 | 0.00048 | 0.00037 | -0.00024-0.00120 |
| 20 | 0.00028 | 0.00030 | -0.00031-0.00087 |
| 21 | 0.00004 | 0.00023 | -0.00041-0.00049 |
| 22 | -0.00015 | 0.00019 | -0.00051-0.00022 |
| 23 | 0.00056 | 0.00023 | -0.00039-0.00050 |
| 24 | 0.00079 | 0.00051 | -0.00020-0.00178 |
| 25 | -0.00020 | 0.00018 | -0.00055-0.00014 |
| 26 | -0.00023 | 0.00019 | -0.00060-0.00014 |
| 27 | -0.00008 | 0.00035 | -0.00077-0.00061 |
